# Bladder Cancer Biomarkers: Recent Advances in Early Detection, Treatment Prediction, and Prognosis

**DOI:** 10.32604/or.2026.086230

**Published:** 2026-09-14

**Authors:** Ziyou Bai, Xiaoyan Song, Jiayin Sun, Wen Xiao, Xiangui Meng, Wei Dong

**Affiliations:** 1 Department of Urology, Union Hospital, Tongji Medical College, Huazhong University of Science and Technology, Wuhan, China; 2 Institute of Urology, Tongji Medical College, Huazhong University of Science and Technology, Wuhan, China; 3 Department of Ultrasound, Traditional Chinese and Western Medicine Hospital of Wuhan, Tongji Medical College, Huazhong University of Science and Technology, Wuhan, China

**Keywords:** Bladder cancer, biomarker, translational urology, liquid biopsy

## Abstract

Bladder cancer (BC) is a prevalent malignancy characterized by a high recurrence rate and the necessity for long-term surveillance demands, creating a need for accurate yet practical tools for early detection and monitoring. While current standards including cystoscopy, urinary cytology, and imaging remain indispensable, their clinical utility is constrained by invasiveness, suboptimal sensitivity for selected lesions or low-grade lesions, inter-observer variability, and cumulative costs. Currently, biomarker research has expanded from single protein assays to multi-analyte strategies encompassing DNA, RNA, proteins, extracellular vesicle-associated cargo, and metabolomics signatures. This review synthesizes recent advances in diagnostic, surveillance, prognostic, and treatment-predictive biomarkers, with emphasis on assay principles, analytical platforms and reported performance where available. Furthermore, we delineate critical barriers to translation, including limited prospective multicenter validation, heterogeneous pre-analytical workflows, inconsistent thresholds, and the persistent trade-off between sensitivity and specificity in real-world benign urologic conditions. Ultimately, while emerging biomarkers and multi-omics panels hold transformative potential as non-invasive adjuncts in risk-adapted clinical pathways, their routine adoption is contingent upon rigorous clinical integration and standardized validation.

## 1 Introduction

Bladder cancer (BC) stands as the tenth most prevalent malignancy globally, with an annual incidence of 573,278 new cases and 212,536 deaths, representing a formidable burden on global healthcare systems [1]. The disease predominantly affects the elderly and male populations, with a male-to-female ratio of approximately 3:1 and a median age at diagnosis of 69 years in men and 71 years in women [2]. Clinically, BC is bifurcated into non–muscle-invasive bladder cancer (NMIBC) and muscle-invasive bladder cancer (MIBC), reflecting profound biological and clinical heterogeneity. Approximately 75% of newly diagnosed cases are NMIBC, which, despite a relatively favorable initial prognosis, is characterized by a recurrence rate of 50–80% and a non-negligible risk of progression to MIBC [3]. MIBC exhibits variable outcomes driven by clinical, pathological and molecular features, and remains associated with substantial morbidity and mortality [4]. Owing to the need for lifelong surveillance and repeated interventions, BC ranks among the most expensive malignancies to manage on a per-patient basis.

Cystoscopy and urinary cytology remain reliable methods for the diagnosis and surveillance of BC. Nevertheless, both approaches are constrained by inherent limitations. Cystoscopy is invasive, costly, and may miss flat lesions such as carcinoma *in situ*, while its sensitivity remains suboptimal despite technical refinements [5]. Although urinary cytology demonstrates high specificity, it requires adequate cellular yield and shows limited sensitivity for low-grade tumors, with diagnostic performance influenced by sampling conditions and subjective interpretation [6]. Imaging modalities such as computed tomography are routinely employed for surveillance, yet their ability to detect minimal residual disease (MRD) or early recurrence is restricted by detection thresholds and measurement variability [7]. Collectively, these shortcomings highlight an unmet clinical need for accurate, non-invasive and cost-effective diagnostic and monitoring strategies in BC.

Biomarkers, defined as measurable biological molecules reflecting pathogenic processes [8], have emerged as promising tools to complement and potentially refine current diagnostic paradigms in BC. Several urinary biomarkers have achieved FDA clearance (as shown in Table 1), including the NMP22 and BTA assays, which demonstrate variable sensitivities across tumor grades, alongside UroVysion fluorescence *in situ* hybridization and the ImmunoCyt test, each exhibiting distinct performance characteristics in terms of sensitivity and specificity.

**Table 1:** FDA approved biomarkers for bladder cancer.

Biomarker (Test)	Target	FDA-Approved Indication
NMP22	Detects nuclear matrix protein 22, released from apoptotic urothelial tumor cells	Diagnosis and surveillance
BTA assays	Detects human complement factor H-related protein. BTA Stat is qualitative, BTA TRAK is quantitative	Monitoring recurrence in combination with cystoscopy
UroVysion	Multicolor FISH detecting aneuploidy of chromosomes 3, 7, and 17, or loss of the 9p21 locus	Diagnosis and surveillance
ImmunoCyt/uCyt+	Immunocytofluorescence assay using three monoclonal antibodies against CEA and sulfated mucin glycoproteins	Surveillance after diagnosis of a primary tumor

**Figure 1:**
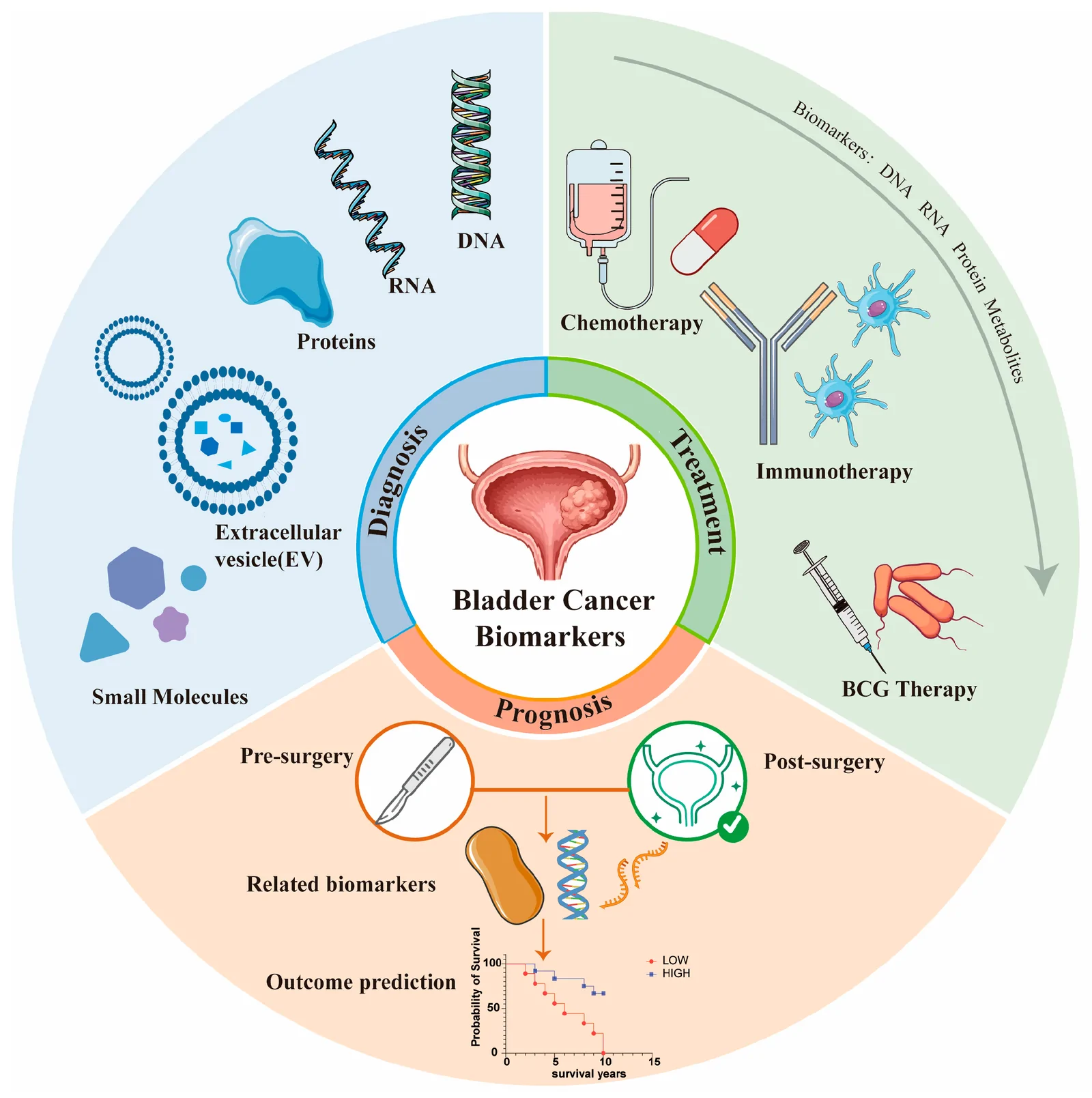
Summary pie chart of bladder cancer (BC) biomarkers. BCG, bacillus calmette–guérin.

Beyond these established assays, increasing attention has been directed toward molecular approaches such as circulating cell-free DNA (cfDNA) analysis and microsatellite instability testing [9], which offer opportunities for minimally invasive detection and dynamic disease monitoring. In parallel, a broad spectrum of genetic, epigenetic, protein-based and exosome-derived markers has been reported, reflecting rapid advances in high-throughput technologies and tumor molecular profiling. However, despite the proliferation of candidate biomarkers, a comprehensive synthesis of the most recent clinical and technical developments is currently lacking. In this review, we summarize the advancements in BC biomarkers for diagnosis, therapeutic prediction and prognosis in the latest three years (as shown in Fig. 1), with a particular focus on emerging molecular assays and delineate current evidence supporting their integration into clinical practice.

## 2 Biomarkers for Diagnosis

### 2.1 DNA-Related Biomarkers

#### 2.1.1 cfDNA

**Figure 2:**
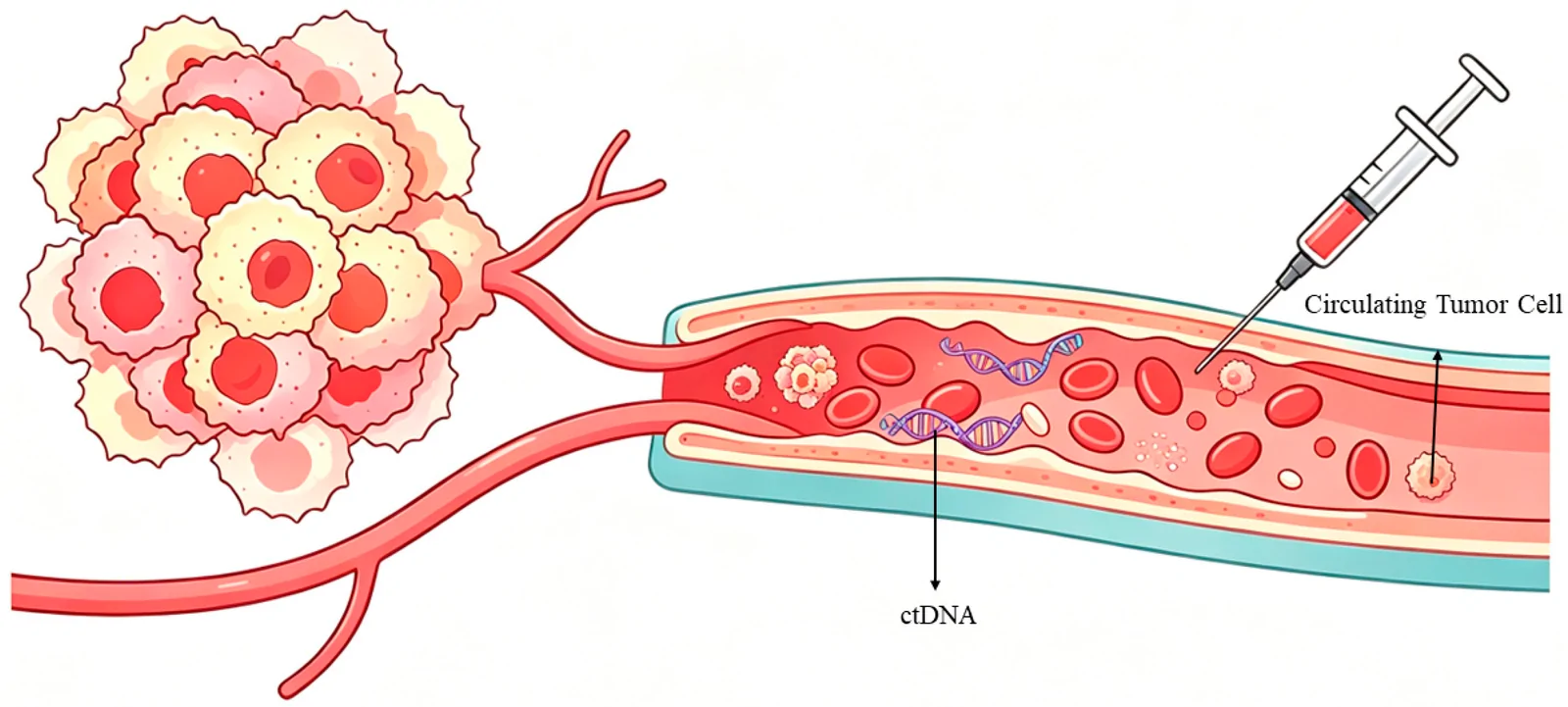
Conceptual illustration of circulating tumor DNA (ctDNA).

There are three extracellular fragmented DNA molecules released into body fluids primarily through apoptosis, necrosis, or active secretion. cfDNA is present in various body fluids including blood, urine, and saliva, serving as a collective term for all extracellular free DNA. Circulating tumor DNA (ctDNA) specifically denotes the tumor-derived fraction of cfDNA in the blood; it acts as a “systemic signal” of tumor burden and reaches higher concentrations in metastatic cancers (as shown in Fig. 2). Urinary tumor DNA (utDNA) exists exclusively in urine, where DNA released from bladder tumors is more readily enriched due to direct contact with urine, making it a unique dedicated reporter for bladder cancer. In healthy individuals, cfDNA originates mainly from hematopoietic cell turnover, whereas in cancer patients, tumor-derived DNA fragments (ctDNA and utDNA) carry tumor-specific genomic and epigenetic features—such as *TP53* mutations and aberrant DNA methylation—thus serving as a molecular fingerprint for disease monitoring [10].

In a large urine-based study including 156 BC patients and 79 matched controls, cfDNA integrity was quantified by qPCR targeting long and short fragments classified by 125 bp of ACTB, AR, MYC, BCAS1 and STOX1. The ACTB integrity ratio increased with tumor stage. The short MYC fragment showed diagnostic value with sensitivity at 50% and a specificity at 95%, improving to 70% sensitivity and 97% specificity in MIBC [11].

Plasma cfDNA methylation was further evaluated in 72 MIBC patients from the prospective SWOG S1314 trial using the Infinium MethylationEPIC array. A methylation response score predicted pathological response to neoadjuvant chemotherapy (NAC) and achieved 79% accuracy when combined with circulating tumor DNA fraction [12].

#### 2.1.2 ctDNA

Serial ctDNA monitoring using Signatera in 23 NMIBC patients detected ctDNA in 35% of cases, facilitating early detection of MRD and influencing treatment decisions [13]. In a cohort of 42 MIBC patients undergoing radical cystectomy, tumor-agnostic droplet digital PCR (ddPCR) targeting TERT and ATM mutations showed prognostic relevance, with ctDNA positivity before surgery and at 4 and 12 months associated with progression [14]. A prospective study of 94 post-cystectomy patients undergoing tumor-informed ctDNA testing showed excellent diagnostic performance, with 100% sensitivity, 91.8% specificity, 84.5% positive predictive value, and 100% negative predictive value compared with imaging [15].

#### 2.1.3 utDNA

For recurrence surveillance, BladMetrix utDNA testing in 47 NMIBC patients showed 91% sensitivity, reducing cystoscopies by 55% [16]. The multidimensional utLIFE assay integrating shallow whole-genome sequencing and targeted sequencing achieved 80.7% sensitivity, 96.2% specificity, and 90.7% accuracy for residual tumor detection, while predicting recurrence-free survival (RFS) [17]. In MIBC bladder-preservation strategies, detectable ctDNA predicted metastasis, while utDNA positivity correlated with shorter bladder-intact survival [18]. utDNA after Bacillus Calmette–Guérin (BCG) induction strongly predicted high-grade recurrence [19]. The RePhyNERX algorithm refined tumor mutation lists using urine, improving MRD specificity [19]. Additionally, Urinary mutation marker M_u_ predicted haematuria residual disease after NAC with sensitivity of 91% and specificity of 50% [20].

#### 2.1.4 DNA Methylation and Mutations, and Novel Detection Technologies

In a prospective multicenter study of 1099 hematuria patients, a single-site PENK methylation assay achieved 89.2% sensitivity and 87.8% specificity for high-grade or invasive BC [21]. Tobacco smoking is a major risk factor for bladder cancer, and emerging evidence suggests that epigenetic alterations, particularly DNA methylation, may serve as promising biomarkers for risk assessment. In a nested case-control study involving the PLCO and ATBC cohorts, researchers examined smoking-related white blood cell methylation markers and their contribution to bladder cancer risk prediction beyond conventional questionnaire-based smoking metrics. Among 2670 previously reported smoking-associated CpG sites, 200 were differentially methylated by smoking status and 28 were significantly associated with bladder cancer risk. The top smoking-related CpG, cg05575921, achieved a predictive performance comparable to classical smoking metrics. Notably, including the first principal component derived from the 200 smoking-related CpGs together with smoking metrics improved prediction, and this component remained significantly associated with bladder cancer risk after adjusting for smoking metrics [22]. Moreover, FGFR3 alterations were analyzed in 389 BLCA tumors; mutation status correlated with molecular subtypes and immune landscape, and LumP/aFGFR3 patients showed higher checkpoint immunotherapy response [23].

Building on these molecular findings, several urine-based DNA methylation and mutation assays have recently been developed as noninvasive tools for bladder cancer diagnosis and surveillance. The UI-Seek test integrates assessments of FGFR3 and TERT mutations together with aberrant methylation of ONECUT2 and VIM to generate a UC-score. In a multicenter prospective trial including 947 participants, UI-Seek achieved a sensitivity of 91.37% and a specificity of 95.09% for detecting urothelial carcinoma. Notably, its sensitivity reached 75.81% for low-grade Ta tumors and exceeded 93% for high-grade Ta and muscle-invasive stages (T1–T4) [24]. Another urinary assay, OncoUrine, evaluates a panel of mutation and methylation biomarkers. For initial diagnosis in patients presenting with hematuria, OncoUrine demonstrated a sensitivity of 80% and a specificity of 91.9%, potentially avoiding 72.3% unnecessary cystoscopies. For recurrence surveillance in NMIBC patients, OncoUrine showed a sensitivity of 100% and a specificity of 68.2%, correctly predicting 80% of eventual recurrences and enabling 62.4% of spared cystoscopies [25].

Several additional urine-based molecular assays have been evaluated for BC detection and surveillance, each targeting different biomarkers. The Uromonitor test is a real-time PCR assay that detects hotspot mutations in TERT promoter (C228T and C250T), FGFR3 (R248C, S249C), and KRAS (G12/13, Q61). Its diagnostic performance has varied across studies. In a large single-center surveillance study for NMIBC recurrence, Uromonitor demonstrated a sensitivity of 87% and a specificity of 99% [26]. However, a subsequent multicenter real-world study reported a lower sensitivity of 49.3% with comparable specificity of 93.3% [27], and an external multicenter validation of Uromonitor-v2 found a sensitivity of only 36% and a specificity of 93% [28], suggesting performance heterogeneity that warrants further investigation. The uTERTpm ddPCR assay detects TERT promoter mutations including C228T, C250T, and rare variants (CC242-243TT, C228A, A161C). In a prospective cohort comparing uTERTpm ddPCR with urine cytology and Uromonitor for primary BC diagnosis, uTERTpm ddPCR achieved the highest sensitivity of 79.7% [29]. The Xpert Bladder Cancer Monitor (XBCM) is a CE-marked test that measures mRNA levels of ABL1, CRH, IGF2, ANXA10, and UPK1B. Cross-sectional studies have reported a sensitivity around 85% and a specificity of around 75% for high-grade NMIBC. In a randomized setting, alternating cystoscopy with XBCM reduced the number of follow-up cystoscopies in high-grade NMIBC patients without compromising recurrence detection [30].

Apart from the aforementioned well-established and commercially available detection assays, including those discussed above and Bladder EpiCheck [31], novel detection methodologies are emerging. For instance, A novel potential-resolved electrochemiluminescence (ECL) immunosensor was developed for simultaneous detection of bladder cancer biomarkers in urine. DNA tetrahedra were used as capture probes, while Ru-MOF@AuNPs and AuAgNCs served as signal reporters, generating well-separated ECL signals for NUMA1 and CFHR1 during a single potential scan. The assay showed good sensitivity, selectivity, and stability, and performed reliably in human urine samples. This multiplex strategy provides a promising noninvasive platform for simultaneous detection of multiple bladder cancer markers and may expand the application of ECL-based bioassays in clinical analysis [32]. The above novel commercially available DNA detection methods are summarized in Table 2.

**Table 2:** Novel commercially available testing methods in DNA-related biomarkers for diagnosis.

Method	Biomarkers	Reported Performance	Reference
UI-Seek	FGFR3, TERT mutations; ONECUT2, VIM methylation	Sn = 91.37%, Sp = 95.09%; Sn = 75.81% for low grade Ta	[24]
OncoUrine	Mutation & methylation panel	Initial: Sn = 80%, Sp = 91.9%;	[25]
		Surveillance: Sn = 100%, Sp = 68.2%	
Uromonitor	TERT (C228T, C250T), FGFR3 (R248C, S249C), KRAS (G12/13, Q61)	Variable Sn: 87% (single-center) to 36–49.3% (multicenter);	[26,27,28]
		Sp: 93–99%	
uTERTpm ddPCR	TERT promoter (C228T, C250T, rare variants)	Sn = 79.7% (highest compared to cytology & Uromonitor in primary diagnosis)	[29]
Xpert Bladder Cancer Monitor (XBCM)	ABL1, CRH, IGF2, ANXA10, UPK1B mRNAs	Sn ≈ 85%, Sp ≈ 75% for high-grade NMIBC; Reduced follow-up cystoscopies in a randomized trial	[30]

### 2.2 RNA-Related Biomarkers

#### 2.2.1 Long Non-Coding RNA (lncRNA)

In BC, the lncRNA BCYRN1 shows oncogenic features: experimental knockdown in BC cell lines reduced proliferation, migration, 3D spheroid formation, and promoted apoptosis, with cell-cycle suppression consistent with G2/M arrest. For clinical translation, BCYRN1 was quantified in serum exosomes, where BC patients had significantly higher levels than healthy donors, and levels dropped after complete resection, supporting its use as a minimally invasive diagnostic indicator and a potential therapeutic target [33].

#### 2.2.2 tRNA-Derived Small RNAs (tsRNAs)

Two circulating tsRNAs (tRF-1:28-chrM.Ser-TGA and tiRNA-1:34-Glu-CTC-1-M2) were reported to be enriched in plasma from BC patients and suggested to originate from bladder tumor tissue rather than other urinary tract conditions. The clinical sampling covered 160 patients collected prior to treatment. Diagnostic performance was assessed by ROC analysis across 228 plasma samples: AUCs were 0.88 (tRF-1:28-chrM.Ser-TGA) and 0.80 (tiRNA-1:34-Glu-CTC-1-M2); a 2-tsRNA panel improved discrimination with AUC 0.93. As a comparator, urine cytology was referenced with specificity of 98.6% and sensitivity of 40.2%, and the tsRNA panel outperformed cytology. Mechanistically, analyses linked predicted targets to lipid-related pathways, and both *in vivo* and *in vitro* assays suggested these tsRNAs could modulate triglycerides/cholesterol/free fatty acids [34].

#### 2.2.3 MicroRNAs (miRNAs)

Tissue-based profiling using TCGA-BLCA identified an 8-miRNA signature comprising five upregulated miRNAs (miR-200a, miR-210, miR-93, miR-130b, miR-455) and three downregulated miRNAs (miR-100, miR-30a, miR-143). This signature effectively distinguished tumor tissue from adjacent normal tissue, with AUC values of approximately 0.95, and was significantly associated with overall survival (OS) [35]. Independent validation in FFPE tumor cohorts spanning pTa low-grade, pT1 high-grade, and MIBC showed miR-138-5p and miR-200a-3p decreased while miR-146b-5p and miR-155-5p increased in MIBC. A 3-miRNA KNN classifier achieved an accuracy of 0.94 and was coupled with conformal prediction to avoid test-set misclassification, supporting miRNA-assisted invasiveness stratification [36]. Additionally, a new noninvasive method used urease-driven magnetic nanomotors to co-detect urinary miRNA-21 and miRNA-182, combining autonomous motion with magnetic enrichment to reach detection limits of 29 fM and 362 fM, respectively, enabling sensitive dual-miRNA quantification in urine [37].

### 2.3 Protein-Related Biomarkers

#### 2.3.1 Protein Biomarkers

Survivin, a small inhibitor-of-apoptosis protein, supports malignant growth by coupling apoptosis resistance with cell-cycle control, which motivated its evaluation in noninvasive testing. A urine-focused meta-analysis reported strong diagnostic performance for both urinary survivin mRNA and protein, indicating high discrimination in urine specimens [38].

Beyond survivin, other urine proteins show varying utility. Urinary LGALS3BP increased with NMIBC grade and, after pooling two cohorts, achieved 66.67% sensitivity and 79.25% specificity. Immunoblotting further suggested tumor-associated size-pattern changes consistent with altered processing [39]. A proteomics-driven workflow identified stage-associated tissue proteins and then tested candidates in urine: NNMT and GALK1 both helped distinguish non-invasive low-grade from invasive high-grade disease, while combining them yielded prognostic accuracy [40]. For invasive risk assessment, SH3YL1 and NOX4 showed limited performance in NMIBC but stronger prediction in MIBC, and low SH3YL1 expression was linked to worse survival, supporting dual diagnostic–prognostic relevance in MIBC [41].

#### 2.3.2 New Detection Methods

Recent advances in multiplex and point-of-care technologies have further expanded the clinical potential of protein-based biomarkers. A real-world 10-plex urinary immunoassay (Oncuria-Detect) evaluated prospectively collected urine from 931 haematuria patients across multiple international centers, along with 69 disease controls. In the independent test set it achieved 85% sensitivity and 72% specificity with similar performance across grades and stages [42]. In addition, A point-of-care optical device that avoids urine preprocessing leveraged urinary hyaluronidases to release fluorophore-carrying particles and enabled smartphone readout. In a double-blind set, it separated cancer (including early-stage and haematuria cases) with approximately 90% accuracy [43].

### 2.4 Extracellular Vesicles (EVs)-Related Biomarkers

EVs are membrane-bound particles released into body fluids that shuttle bioactive cargo and can influence tumor biology, such as signaling, invasiveness, and angiogenesis. In liquid biopsy practice, EVs are commonly discussed as a mixed population because current isolation methods cannot perfectly separate biogenesis routes; mechanistically, they are often grouped by diameters as exosomes, microvesicles, and apoptotic bodies [44,45,46].

#### 2.4.1 Newly Found Biomarkers

Within urinary EVs (uEVs), protein features have been proposed for BC detection and risk stratification: panels including MASP2, C3, A2M, CHMP2A and NHE-RF1 were highlighted as having stronger discrimination for first diagnosis, while other proteins, such as HBB and HBA1, showed differences in recurrence settings, although their biological significance remains less clear [47]. In addition, uEV protein signatures appeared to reflect muscle invasiveness, with MCP-1, PDCD1, TIE2 and TNFRSF9 significantly enriched in MIBC-derived EVs [48]. Beyond protein identity, uEV glycosylation profiling across 333 individuals mapped 252 N-glycans and suggested decreased fucosylation with increased sialylation in BC [49].

For uEV mRNAs, a 3-transcript panel (SRGN, FLI1, MACROH2A2) derived from urinary EVs achieved high AUC for early-stage BC and decreased after surgery, supporting tumor-associated shedding and potential utility for noninvasive monitoring [50].

EV-associated lipids also show promise: urine EV–linked neolactotetraosylceramide was elevated in BC with accuracy of 82%, then supported by ELISA in discovery and multicenter validation, where MIBC showed accuracy of 64%, and levels tracked severity [51].

#### 2.4.2 New EV-Enabled Detection Technologies

A range of innovative technologies has been developed to enhance EV-based biomarker detection. A multispectral three-dimensional DNA machine using aptamer recognition of five uEV protein markers with multimodal ML reached 95.0% accuracy [52]. MXene-enabled nanostructure mass spectrometry platforms allow rapid urinary exosome capture and metabolic profiling, demonstrating high diagnostic performance for tumor stage and subtype classification [53]. Similarly, magnetic ZIF-8 (magMZIF-8)–based EV isolation combined with LC–MS metabolomics and machine learning achieved an AUC exceeding 0.844 [54]. A Pt@CP nanozyme-based immunoassay multiplexing uEV proteins reported promising detection ability [55]. Additional platforms include metal-organic frameworks (MOF)-on-MOF cyclic enrichment chip supporting NORAD-based urinary models, as well as microfluidic herringbone and photonic crystal barcode systems for multiplex exosomal protein detection [56,57].

Conventional ultracentrifugation, while widely used, is time-consuming, yields exosomes with relatively low recovery and purity, and co-pellets protein aggregates and other extracellular vesicles, thereby confounding downstream RNA and protein analyses. In contrast, the magnetic 3D ordered macroporous MOF material (magMZIF-8) described herein exploits a triple mechanism—affinity between metal-oxygen units and phospholipid phosphate groups, electrostatic interactions, and size-exclusion through its 150 nm macropores—to selectively capture exosomes while excluding larger urinary debris. This design reduces contamination from cellular fragments and non-exosomal vesicles, resulting in exosomes with more uniform particle sizes and a concentrated size distribution. Consequently, the extracted RNA and protein cargo more faithfully reflect the exosomal compartment. Similarly, the MOF-on-MOF cyclic enrichment chip (Zr-MOF@Fe-MOF integrated into an ExCE-chip) employs asymmetric impinging streams to enhance collision probability, enabling rapid batch enrichment from volumes exceeding 10 mL. By markedly shortening the processing time (<30 min) and obviating ultracentrifuges, this approach limits RNA degradation and preserves cargo integrity, which is particularly important for labile long non-coding RNAs such as NORAD.

### 2.5 Metabolomics-Derived Small Molecules for Detection

Because lipids are core membrane components and key regulators of energy storage and signaling, altered lipid metabolism can mirror BC biology. In a urine-metabolomics comparison of BC patients with non-cancer controls, 51 discriminative metabolites were reported, with lipid derivatives forming a major subgroup. Ten lipids, including medium-chain fatty acids, acylcarnitines, long-chain fatty acids, and a hydroxy fatty acid, were higher in controls, whereas oleamide was markedly higher in BC and emerged as the single most distinguishing compound. Oleamide, along with isostearic acid and azelaic acid, achieved AUC values exceeding 0.9 [58].

Plasma amino acids can reflect systemic metabolic reprogramming related to nitrogen handling and arginine pathways. A case–control study recruited newly diagnosed BC and controls, measured fasting plasma amino acids by LC–MS, and cross-validated pathway signals using GEO GSE13507. Eleven amino acids differed. After adjustment, ornithine was associated with reduced risk, whereas methionine, arginine, and glutamate were linked to increased risk. A four-amino-acid panel achieved an AUC of 0.864 [59].

Urinary volatile organic compounds (VOCs) integrate downstream metabolic changes and can be modeled for noninvasive detection. Using HS-SPME/GC-MS, VOCs from 87 BC and 90 matched controls were profiled. Machine learning models trained on 27 VOCs achieved strong performance, with a random forest model reaching an AUC of 0.913. An optimized eight-VOC panel further improved validation performance, achieving 89% sensitivity and 92% specificity [60]. However, the diagnostic utility of these VOCs is critically dependent on their chemical stability, as degradation or differential volatilization can occur during sample storage and processing. Factors such as storage temperature, freeze-thaw cycles, and prolonged room temperature exposure have been shown to significantly alter VOC profiles, highlighting the urgent need for standardized protocols for urine collection, handling, and storage to ensure reliable clinical translation [61].

A label-free SERSomes approach profiles urine metabolite spectral patterns via surface-enhanced Raman scattering (SERS) and applies machine learning for classification, aiming for rapid, noninvasive, low-cost screening. Reported performance included low-grade bladder cancer diagnosis accuracy of 89.47% and stratification accuracy of 90%. The 749–767 cm^−1^ spectral region contributed most prominently to the classification model and was mainly assigned to tryptophan and ethanolamine, suggesting that altered tryptophan metabolism may be closely associated with bladder carcinogenesis, the establishment of an immunosuppressive tumor microenvironment, and disease progression. For the discrimination between T1 and T2 stage patients, the 1440–1461 cm^−1^ band showed the highest contribution, which may correspond to deoxyribose derived from nucleic acids and CH2 deformation vibrations from lipids, indicating substantial differences in lipid metabolic profiles between these two stages. Collectively, these findings support the potential of this approach as a promising urinary biomarker-based tool for bladder cancer detection and stratification [62].

## 3 Biomarkers for Treatment

BC management relies heavily on disease stage. For non-muscle-invasive BC, intravesical BCG serves as the standard of care. As the disease progresses to MIBC, cisplatin-based NAC prior to radical cystectomy becomes a commonly used treatment modality, significantly improving OS [63]. Additionally, immune checkpoint inhibitors (ICIs) have recently revolutionized the treatment landscape for advanced and metastatic cases [64,65]. Despite these clinical advances, predicting patient response remains a critical hurdle. Approximately 30–50% of patients fail BCG therapy [66], nearly half exhibit resistance to NAC [67], and responses to immunotherapy vary widely. Because current clinical parameters cannot reliably identify which patients will benefit, discovering robust predictive tools is urgently needed. To address these distinct clinical challenges, our subsequent discussion on treatment-predictive biomarkers is fundamentally categorized into three primary therapeutic pillars: chemotherapy, immunotherapy, and BCG.

### 3.1 Chemotherapy-Related Biomarkers

#### 3.1.1 DNA-Related Biomarkers

##### ctDNA

ctDNA dynamics serve as a valuable tool for predicting chemotherapy efficacy in MIBC. Post-cystectomy ctDNA analysis demonstrates high accuracy in detecting metastatic recurrence, achieving 94% sensitivity and 98% specificity [68]. Similarly, whole-genome sequencing of ctDNA can predict NAC prognosis with a 131-day lead time over imaging, and it detects postoperative recurrence with 91% sensitivity and 92% specificity [69].

##### Other Genomic Biomarkers

Genomic profiling helps stratify NAC responders. Genomic proteomic analysis of transurethral resection samples revealed that basal-like subtypes exhibit higher pathological complete response (pCR) rates compared to the cytoskeleton-active luminal subtype. Using targeted panels on 105 pre-NAC specimens, mutations in ATM, RB1, FANCC, or ERCC2 predicted cystectomy (pT0) status with an 86% negative predictive value and 48% positive predictive value [70]. An independent cohort of 165 patients later validated that ERCC2 deleterious mutations positively correlate with NAC response, though the other three genes were not confirmed [71]. Further genomic analyses revealed that amplifications at chromosome 6p22.3 (including E2F3 and SOX4) were associated with improved treatment response, whereas FGFR3 mutations were linked to reduced responsiveness [72]. Additionally, fluorescence *in situ* hybridization (FISH) and IHC analyses of 302 MIBC and 63 metastatic specimens showed that MTAP loss, functioning as a surrogate for CDKN2A homozygous deletion with 84.0% sensitivity and 96.3% specificity, correlates with FGFR3 mutations and the luminal-URO subtype [73]. Moreover, an 8-gene signature (IL32, AHNAK, ANXA5, FN1, GSN, CNN3, FXYD3, CTSS) has been proposed for risk stratification, where high-risk scores indicate an immunosuppressive microenvironment and significant cisplatin resistance [74].

#### 3.1.2 RNA-Related Biomarkers

RNA-based markers also influence chemosensitivity. The lncRNA JHDM1D-AS1 is involved in promoting tumor growth and stimulating angiogenesis. In a clinical cohort of 30 fresh bladder cancer tissues, its expression correlated positively with the epigenetic regulator JHDM1D gene. Notably, silencing JHDM1D-AS1 combined with gemcitabine treatment significantly enhanced cytotoxicity, increased cell death rates, and induced cell cycle arrest, suggesting its potential to overcome chemotherapy resistance [75].

#### 3.1.3 Protein-Related Biomarkers

Protein biomarkers offer critical insights into chemotherapy sensitivity. Cyclin-dependent kinase 6 (CDK6), a crucial cell cycle regulator, was evaluated in a retrospective cohort of 933 MIBC patients. High CDK6 expression was correlated with poor prognosis but favorable responses to platinum-based chemotherapy. Integrating CDK6 with PD-L1 and tumor mutation burden (TMB) established a robust predictive scoring model [76,77]. Similarly, high GREM1 expression indicates sensitivity to cisplatin and other chemotherapeutics [78]. Advanced proteomic profiling further identifies specific resistance patterns. Mass spectrometry of 88 MIBC samples revealed a specific non-responder subtype characterized by high NECTIN4 and Her2 expression [79]. Furthermore, a multi-omics analysis of 60 MIBC tumors identified a short ATAD1 isoform and members of the RAF protein family as markers of chemosensitivity, while highlighting Wnt and JAK/STAT pathways as potential targets to overcome chemoresistance [80].

#### 3.1.4 Metabolomics-Derived Small Molecule Biomarkers

Other biomarkers, particularly metabolomic profiles, serve as emerging predictive tools. A pilot study utilizing nuclear magnetic resonance spectroscopy longitudinally monitored 14 BC patients undergoing platinum-based chemotherapy. The analysis detected significant baseline elevations and treatment-induced perturbations in glycolysis, purine, and amino acid metabolism. Notably, trimethylamine N-oxide, a metabolite fostering inflammation and oxidative stress, was elevated at baseline but decreased throughout the chemotherapy course, highlighting its potential for monitoring and predicting treatment response [81].

### 3.2 Immunotherapy-Related Biomarkers

#### 3.2.1 ctDNA

ctDNA can act as a marker of MRD after cystectomy, enriching for patients at high recurrence risk who may benefit from adjuvant immunotherapy. In an earlier exploratory analysis, the phase III IMvigor010 trial demonstrated that baseline ctDNA positivity identified patients with markedly inferior prognosis under observation (HR = 6.3) and significant OS benefit from adjuvant atezolizumab (HR = 0.59). On-treatment ctDNA dynamics further refined prognostication. A greater reduction in ctDNA levels by cycle 3 day 1 correlated with progressively longer OS, with median OS of 60.0 months for complete clearance, 34.3 months for over 50% reduction, and 19.9 months for below 50% reduction [82]. Later, in the phase III double-blind IMvigor011 trial, patients without radiographic disease underwent serial ctDNA monitoring for up to 1 year, and ctDNA-positive cases were randomized to atezolizumab and placebo every 4 weeks for 1 year. Among 250 randomized ctDNA-positive patients, atezolizumab prolonged disease-free survival (DFS) with a median of over 5 months and OS with a median of over 10 months. Persistently ctDNA-negative patients were observed without study drug and showed high DFS with 95% at 1 year and 88% at 2 years, indicating ctDNA can be a promising guide for DFS and OS for patients receiving atezolizumab [83].

In another phase III study, CheckMate 274, 709 high-risk muscle-invasive urothelial carcinoma patients were randomized to nivolumab 240 mg q2w and placebo under 1 year after radical surgery. In a post hoc cohort with baseline ctDNA measured by Signatera (133 assessable; 40.6% ctDNA-detectable), detectable ctDNA marked very poor DFS at only 5.0 median months compared with undetectable ctDNA at 52.1 median months. Treatment effect appeared enriched in ctDNA-detectable disease but not in ctDNA-undetectable cases [84]. Separately, adaptive-immunity features, such as high IFN-γ signature, CD4 expression and CD8 infiltration, contributed to composite models predicting improved outcomes on nivolumab [84].

#### 3.2.2 Other Genomic Biomarkers

Enfortumab vedotin efficacy in metastatic urothelial carcinoma (mUC) is closely linked to specific genomic alterations. NECTIN4 amplification, evaluated via FISH in a 108-patient cohort, was found in approximately 26% of mUC cases. This amplification strongly predicted Enfortumab vedotin response, yielding an objective response rate of over 90% and reducing the risk of death by 92% [85,86]. Additionally, immunohistochemistry analysis of 28 mUC patients revealed that tumors exhibiting abnormal p53 and low FGFR3 expression demonstrated significantly better therapeutic responses to Enfortumab vedotin [87].

Anoikis is a specific form of apoptosis triggered when cells detach from the extracellular matrix, preventing improper cell re-attachment; its resistance drives cancer progression [88]. A study led by Xie et al. formulated a four-gene signature Ascore, based on four anoikis-related signatures (CERCAM, EMP1, GNLY, and PTPRR) to predict immunotherapy responses, which was better than PD-L1 [89]. Similarly, two anoikis subgroups were identified utilizing 17 anoikis-related genes. Cluster B had higher gene expression and demonstrated lower sensitivity to immunotherapy [90].

The SWItch/sucrose non-fermentable (SWI/SNF) chromatin-remodeling complex fundamentally regulates DNA transcription and repair. Alterations in its subunits occur in over 40% of bladder cancers, with the highest frequencies in ARID1A, ARID1B, ARID2, SMARCA4, and PBRM1. Patients harboring these mutations were correlated with elevated TMB and improved survival following immune checkpoint blockade (ICB), with genotype models achieving an AUC of 0.909 in mutant tumors [91]. These findings have been validated in a phase 2 trial of 45 patients receiving atezolizumab plus radiotherapy; genomic alterations in CDK12, GNAS, NOTCH2, and ARID1A correlated strongly with a high pCR rate [92]. Furthermore, a derived SWI_SNF_Score accurately predicted TMB phenotypes and treatment sensitivities [93].

Immune-related gene signatures are emerging as promising biomarkers for prognostic stratification and therapeutic guidance in BC. Integrative bioinformatic analysis identified eight significantly downregulated genes (LIMS2, TP53INP2, IRAK3, STX2, CYP27A1, IL11RA, KCNMB1, and PDLIM7) which were mainly enriched in focal adhesion–related pathways, highlighting a potential link between immune adhesion signaling and bladder cancer progression; all eight genes showed diagnostic value with an AUC over 0.7 [94]. In the context of immunotherapy prediction, a machine learning–derived 49-gene signature developed from the IMvigor210 cohort achieved an AUC of 0.75 in an independent mUC dataset and outperformed several established molecular and tumor microenvironment signatures, suggesting superior utility for predicting response to immune checkpoint inhibitors [95]. In addition, a risk model derived from differentially expressed genes between the efferocytosis subtypes, which ultimately comprised four key genes (SERPINE2, DPYSL3, CTSE, and KRT16), effectively stratified patients into high- and low-risk groups. The high-risk subgroup exhibited worse survival, greater immune infiltration, and increased immune checkpoint expression, indicating a population potentially more likely to benefit from immunotherapy [96]. Furthermore, an angiogenesis-associated gene scoring (AAG_score) system established by Cao and Zheng identified immune-active and prognostically favorable tumors with low AAG_score, characterized by higher microsatellite instability, stronger mutational tendency, and enhanced immune activation, further supporting the relevance of immune-related transcriptomic features as biomarkers in bladder cancer. Importantly, subsequent cellular functional assays in the same study confirmed that NID2, a pivotal gene incorporated into this AAG_score, significantly promotes bladder cancer cell proliferation, providing experimental evidence for the biological validity of this scoring system [97].

Moreover, in NMIBC, whole-exome and RNA sequencing of 81 samples identified four genomic subtypes. Notably, the Aristolochic acid and genome instability subtypes demonstrated significantly better outcomes following immunotherapy compared to the FGFR3/HRAS subtype [98].

### 3.3 BCG-Related Biomarkers

In high-risk NMIBC treated with intravesical BCG, a pre-existing Th2-skewed tumor microenvironment may predict benefit: on pretreatment biopsies, immunohistochemistry quantified the GATA3+/T-Bet+ ratio and EPX+ eosinophil density and degranulation, summarized as a “Th2-score”. A cutoff over 48.1 identified responders with 91% sensitivity, while specificity was reported as low [99]. Separately, targeted multigene sequencing of pretreatment transurethral resection of bladder tumor tissues in 19 high-grade NMIBC patients found TP53 and FGFR3 as the most frequent mutations; responders more often harbored mutations including tyrosine-kinase receptor genes and CTNNB1, whereas non-responders mainly showed TP53 alterations [100]. Beyond single markers, the CHAI platform provides an AI-augmented histology biomarker to guide regimen choice: among treatment-naïve high-grade NMIBC, CHAI-positive patients had inferior high-grade RFS with BCG versus sequential intravesical gemcitabine/docetaxel, while CHAI-negative patients showed no clear difference, supporting treatment-dependent risk stratification [101].

## 4 Biomarkers for Prognosis

### 4.1 DNA-Related Biomarkers

#### 4.1.1 ctDNA

Across multiple bladder cancer cohorts, ctDNA functions as a minimally invasive marker of MRD and relapse risk. In a 112-patient analysis, pre–radical cystectomy ctDNA positivity associated with inferior RFS and higher odds of nodal involvement and locally advanced disease; ctDNA detected pre-RC and during the MRD window independently predicted recurrence [102]. Furthermore, Sfakianos et al. reported that postoperative ctDNA detectability during the MRD window and surveillance correlated with shorter DFS [103]. A prospective perioperative study using the AVENIO ctDNA platform tracked common alterations, notably TERT and TP53, and found postsurgery ctDNA negativity linked to longer RFS; conversion from ctDNA-positive to ctDNA-negative after surgery aligned with longer median survival of 18 months [104]. Longitudinal follow-up in NAC-treated and NAC-naïve cohorts demonstrated that post-RC ctDNA identified metastatic relapse with 94% sensitivity and 98% specificity, and ctDNA was prognostic both before and after RC in NAC-naïve patients [68]. In stage III mUC treated with ipilimumab plus nivolumab, absence of plasma ctDNA associated with pathological pCR and longer progression-free survival (PFS), while urinary ctDNA was only correlated with pCR [105].

#### 4.1.2 Other Genomic Biomarkers

Several DNA-derived gene programs link tumor biology with prognosis and immunotherapy context. A 17-gene reactive oxygen species (ROS)–related signature built from TCGA data predicted OS, with AKR1B1 experimentally supporting tumor cell growth phenotypes [106]. Integrative multi-database bioinformatics identified CXCL12 as a shared BLCA–diabetes gene tied to changed immune cell role and tumor traits during high blood sugar states, suggesting metabolic–immune crosstalk [107]. A lactylation-based model connected tumor metabolism to immune-cell associations, which can predict survival outcomes in BLCA patients [108]. A 6-gene prognostic model (CCDC80, NIBAN1, CSPG4, PDGFRA, MAP1A, PCOLCE2) showed robust nomogram discrimination, and linked risk to immune–stromal features, notably NK cells [109]. Du et al. proposed a senescence-related signature comprising BMP6, FN1, CD274, HOXB5, and PPIL3 to predict survival and immunotherapy benefit in bladder cancer. Notably, PPIL3 suppresses tumor growth by inducing cellular senescence [110]. Separately, a study led by Cai et al. using LASSO-COX regression analysis showed a cisplatin-sensitivity gene model highlighted SCAMP2 and reported 1-year AUCs of 0.825 [111]. A calcium-signaling 6-gene signature (ATP2B4, BDKRB2, EDNRA, PDGFRA, EGFR, ADCY7) associated risk groups with TMB, immune infiltration, and predicted therapy responsiveness, with ATP2B4 mechanistically linked to apoptosis via Ca^2+^ flux [112]. Specifically, ATP2B4 is a member of the plasma membrane calcium ATPase family and is responsible for maintaining low cytosolic calcium concentrations by actively transporting Ca^2+^ ions from the cytoplasm to the extracellular environment. Whilst ATP2B4 is significantly overexpressed in bladder cancer tissues, its downregulation results in elevated cytoplasmic Ca^2+^ concentrations, which in turn activate the VDAC1/MCU pathway, through which Ca^2+^ is redirected from the cytoplasm into mitochondria. The ensuing mitochondrial calcium overload ultimately triggers apoptosis in BLCA cells [112].

### 4.2 RNA-Related Biomarkers

Using three public datasets including bladder urothelial carcinoma (BUC) (GSE13507, GSE8358) and Upper Tract Urothelial Carcinoma (Baylor-MDACC) and immunohistochemical analysis in an in-house tissue microarray cohort BUC (“SNUH_TMA”) comprising 226 patients, high TUBB6 mRNA expression was significantly associated with poor OS. Furthermore, functional assays demonstrated that TUBB6-depleted BUC cell lines exhibited markedly reduced migration and invasion abilities in both 2D invasion and 3D spheroid invasion assays [113].

Circular RNAs are covalently closed transcripts that can modulate gene expression and are being explored as prognostic markers in BC; in reported clinical profiling, elevated circ-ZFR expression was associated with worse OS [114].

lncRNAs influence transcriptional and post-transcriptional regulation, which may reflect aggressive tumor biology [115]. Low lnc-GAS5 and high lnc-TUG1 were each linked to poorer OS in BC [115]. In a separate prognostic modeling study, disulfidptosis-related lncRNAs were mined and assembled into a five-lncRNA risk index (AC005840.4, AC010331.1, AL021707.6, MIR4435-2HG, ARHGAP5-AS1), with qPCR used for confirmation; high-risk status mapped to pathways tied to PI3K–Akt signaling, extracellular matrix organization, and immune escape, and the model also suggested higher predicted sensitivity to sorafenib, oxaliplatin, and MK-2206 in high-risk patients [116].

The reported expression-outcome associations in BC showed that higher miR-21 and miR-222 tracked with inferior OS, higher miR-155 and miR-143 tracked with poorer PFS, and lower miR-214 tracked with worse RFS [114].

In the context of immunotherapy, for ICI-treated cohorts, a transcriptomic bladder cancer gene signature derived from bulk and single-cell datasets stratified OS and emphasized antigen presentation and CD8^+^ T-cell activation pathways. Moreover, patients with a concurrently low transcriptomic bladder cancer gene signature score and low TMB exhibited an increased risk of death [117].

### 4.3 Protein-Related Biomarkers

Protein biomarkers provide additional prognostic insights and may also inform therapeutic strategies. Glycyl-tRNA synthetase 1 (GARS1) is reported to be highly expressed in BLCA and associated with worse survival. Integrative analyses further linked GARS1 to immune infiltration patterns and mismatch repair–related genes, and *in vitro* assays supported pro-tumor effects [118]. Adhesion-regulating molecule 1 is consistently upregulated in bladder cancer versus adjacent tissue and predicts inferior OS, whose expression correlated with key immune checkpoints as well as higher CD8^+^ T-cell and macrophage infiltration, suggesting utility not only for prognosis but also for anticipating immunotherapy response patterns [119]. LIM domain binding protein 2 (LDB2) behaves as a metastasis-suppressive, immune-linked marker with a diagnostic discrimination of 0.91 AUC. Mechanistic work connected LDB2 to MAPK signaling and immune cell infiltration correlations [120]. A study led by Smolka analyzed 175 muscle-invasive BC samples; multivariate Cox regression analysis showed CC motif ligand 5 (CCL5) with compartment-specific prognostic value: CCL5 positivity in tumor cells associated with shorter disease-specific survival, whereas lack of CCL5 in immune cells marked worse RFS and identified patients more likely to benefit from adjuvant chemotherapy, with noted exceptions by nodal subgroup [121].

Beyond immune markers, several proteins emerged as promising biomarkers. Baseline plasma PTX3 levels were elevated in patients who subsequently developed metastasis and were associated with muscle-invasive disease, suggesting potential prognostic utility, although further validation is needed [122]. At the systemic level, a meta-analysis showed elevated C-reactive protein (CRP) consistently associates with worse OS and RFS, supporting CRP as a broadly accessible prognostic indicator [123]. Finally, in the Ruijin cohort (64 patients undergoing cystectomy), the mean survival time was 48.26 months in the FOXF1-high group and 28.50 months in the FOXF1-low group. The association of high FOXF1 expression with favorable clinical outcomes was validated in external datasets including GSE48075, GSE13507, GSE31684, IMvigor210, and GSE169455. Moreover, univariate Cox regression analysis demonstrated that FOXF1 expression level (HR = 0.23, 95% CI: 0.085–0.64, *p* = 0.0049) was a prognostic factor. Mechanistic studies suggest that FOXF1 promotes apoptosis via caspase-3 signaling, supporting its role as a protective factor [124].

## 5 Discussion

Although tissue biopsy remains the gold standard for tumor diagnosis owing to its high standardization, consistent results, and accuracy, it is limited by its invasive nature, inability to sample the highest-risk regions, and the fact that the acquired molecular information is heavily influenced by tumor heterogeneity and reflects only the sampled site. In contrast, liquid biopsy, including the analysis of ctDNA, utDNA, and other analytes, offers minimal invasiveness, low risk, repeatable sampling, and suitability for dynamic monitoring, thereby partially overcoming the impact of spatial heterogeneity. However, current liquid biopsy approaches still face challenges such as a lack of laboratory standardization and the need to improve diagnostic accuracy. They cannot fully capture the complexity of a tumor and therefore cannot replace tissue biopsy. In bladder cancer, a complementary strategy is particularly promising: well-established tissue-based pathological markers (e.g., MTAP loss as a surrogate for CDKN2A deletion, and GREM1 expression) can provide definitive initial diagnosis and risk stratification, while multi-omics liquid biopsy panels can be integrated into risk-adapted clinical pathways as non-invasive adjuncts for longitudinal surveillance, detection of MRD, and early identification of resistance. The synergistic use of tissue and liquid biopsies thus enables a more comprehensive understanding of tumor biology and supports personalized, stage-adapted clinical decision-making.

For such a complementary approach to be clinically meaningful, however, the selection and validation of liquid biopsy biomarkers must be governed by rigorous criteria of clinical utility. Guided by the 2022 WHO consensus [125], the 2005 International Consensus Panel on Bladder Tumor Markers [126], and the clinical characteristics of BC, it is evident that an ideal biomarker should serve as a measurable biological surrogate that anticipates clinically meaningful outcomes, such as recurrence or progression, that are otherwise difficult to capture. Consequently, the value of a biomarker must be adjudicated based on its clinical utility rather than scientific novelty alone.

A recurring limitation across urine-marker research is the shortage of high-quality, prospective evidence [127]. Many investigations remain retrospective case–control comparisons that can estimate how many tumors are detected or missed, but do not reliably demonstrate how a marker should be embedded into a real cystoscopy-based decision pathway. As a consequence, external validity is often weakened by heterogeneous designs, variable inclusion or exclusion criteria, incomplete reporting of sampling procedures, and inconsistent analytical thresholds. These factors collectively impede rigorous head-to-head comparisons and robust meta-level synthesis.

A second critical gap is the lack of mechanistic depth. While mechanistic hypotheses can strengthen biological plausibility and help define the most informative patient subgroups, many candidate markers are still supported mainly by association data, and translational work, such as animal models or functional validation beyond cell assays, is frequently incomplete. Third, the trade-off between sensitivity and specificity remains a central challenge. An ideal urine biomarker should provide high sensitivity alongside robust specificity across various benign urological conditions, while remaining rapid and low-cost. In practice, few candidates satisfy all these requirements simultaneously. This challenge is especially relevant if the clinical goal is to reduce cystoscopy frequency, because very high sensitivity is generally required, yet performance in low-grade disease often remains insufficient for replacement of standard surveillance.

Clinically, biomarker selection should be scenario-driven rather than one-size-fits-all. For early detection, sensitivity must be balanced against an acceptable false-positive burden to limit overdiagnosis and downstream procedures. Conversely, for recurrence monitoring, the risk of “missing” disease is the primary concern, making high sensitivity the priority. Given that no single marker is universally optimal, rational strategies should involve standardized multi-marker panels coupled with transparent computational cutoffs, cost-effectiveness evaluation, and multicenter validation that reflects real-world case-mix and pre-analytical variability. Overall, the field is progressively shifting from single-protein assays toward multiplex molecular tests enabled by advances in genomic and transcriptomic technologies. Nonetheless, clinical implementation should be paced by rigorous prospective trials, harmonized quality control, and clear guidance on which markers or panels fit which clinical use cases.

## 6 Conclusions

The development of robust biomarkers for the early detection and surveillance of BC holds transformative potential to improve patient outcomes while alleviating the physical and economic burdens of repeated invasive procedures. There is a systemic trend from single-marker assays toward multi-target combinations, and from conventional signal readouts to highly sensitive and intelligent analytical approaches. Although numerous urine- and blood-based candidates including protein assays, RNA panels, and DNA mutation tests have shown encouraging performance, most remain constrained by variable sensitivity in low-grade disease, limited specificity in benign urologic conditions, and inconsistent reproducibility across cohorts.

To accelerate clinical translation, future research should prioritize well-designed, multicenter prospective trials characterized by standardized pre-analytical handling, transparent analytical thresholds, harmonized reporting of sensitivity and specificity, and predictive values in clearly defined clinical scenarios. Furthermore, mechanistic studies are essential to strengthen biological plausibility and guide the rational combination of biomarkers. Ultimately, while biomarkers are unlikely to fully replace cystoscopy in the immediate future, they represent invaluable, cost-effective adjuncts for refining risk stratification, facilitating early diagnosis, and enabling a more personalized approach to recurrence monitoring for patients with BC.

## Data Availability

Not applicable.

## Abbreviations

The following abbreviations are used in this manuscript:

BC	bladder cancer
NMIBC	non-muscle-invasive bladder cancer
MIBC	muscle-invasive bladder cancer
cfDNA	circulating cell-free DNA
ctDNA	circulating tumor DNA
utDNA	urinary tumor DNA
ddPCR	droplet digital PCR
NAC	neoadjuvant chemotherapy
RFS	recurrence-free survival
BCG	Bacillus Calmette-Guérin
ECL	electrochemiluminescence
lncRNA	long non-coding RNA
tsRNAs	tRNA-derived small RNAs
miRNAs	microRNAs
OS	overall survival
EVs	extracellular vesicles
uEVs	urinary extracellular vesicles
VOCs	volatile organic compounds
SERS	surface-enhanced Raman scattering
ICIs	immune checkpoint inhibitors
pCR	pathological complete response
FISH	fluorescence *in situ* hybridization
TMB	tumor mutation burden
mUC	metastatic urothelial carcinoma
DFS	disease-free survival
ICB	immune checkpoint blockade
PFS	progression-free survival
MRD	minimal residual disease
RC	radical cystectomy
ROS	reactive oxygen species
CRP	C-reactive protein

## References

[1] Sung H , Ferlay J , Siegel RL , Laversanne M , Soerjomataram I , Jemal A , et al. Global cancer statistics 2020: GLOBOCAN estimates of incidence and mortality worldwide for 36 cancers in 185 countries. CA Cancer J Clin. 2021; 71( 3): 209– 49. doi:10.3322/caac.21660. 33538338

[2] Siegel RL , Miller KD , Jemal A . Cancer statistics, 2018. CA Cancer J Clin. 2018; 68( 1): 7– 30. doi:10.3322/caac.21442. 29313949

[3] Cumberbatch MGK , Jubber I , Black PC , Esperto F , Figueroa JD , Kamat AM , et al. Epidemiology of bladder cancer: A systematic review and contemporary update of risk factors in 2018. Eur Urol. 2018; 74( 6): 784– 95. doi:10.1016/j.eururo.2018.09.001. 30268659

[4] Crupi E , de Padua TC , Marandino L , Raggi D , Dyrskjøt L , Spiess PE , et al. Circulating tumor DNA as a predictive and prognostic biomarker in the perioperative treatment of muscle-invasive bladder cancer: A systematic review. Eur Urol Oncol. 2024; 7( 1): 44– 52. doi:10.1016/j.euo.2023.05.012. 37330413

[5] Witjes JA , Bruins HM , Cathomas R , Compérat EM , Cowan NC , Gakis G , et al. European association of urology guidelines on muscle-invasive and metastatic bladder cancer: Summary of the 2020 guidelines. Eur Urol. 2021; 79( 1): 82– 104. doi:10.1016/j.eururo.2020.03.055. 32360052

[6] Hentschel AE , Beijert IJ , Bosschieter J , Kauer PC , Vis AN , Lissenberg-Witte BI , et al. Bladder cancer detection in urine using DNA methylation markers: A technical and prospective preclinical validation. Clin Epigenet. 2022; 14( 1): 19. doi:10.1186/s13148-022-01240-8. PMC8818199 35123558

[7] Alfred Witjes J , Max Bruins H , Carrión A , Cathomas R , Compérat E , Efstathiou JA , et al. European association of urology guidelines on muscle-invasive and metastatic bladder cancer: Summary of the 2023 guidelines. Eur Urol. 2024; 85( 1): 17– 31. doi:10.1016/j.eururo.2023.08.016. 37858453

[8] Dalton WS , Friend SH . Cancer biomarkers—An invitation to the table. Science. 2006; 312( 5777): 1165– 8. doi:10.1126/science.1125948. 16728629

[9] Reynolds T , Bertsche K , Moon D , Moon C . Qualification of the microsatellite instability analysis (MSA) for bladder cancer detection: The technical challenges of concordance analysis. Int J Mol Sci. 2023; 25( 1): 209. doi:10.3390/ijms25010209. PMC10779061 38203379

[10] Lindskrog SV , Strandgaard T , Nordentoft I , Galsky MD , Powles T , Agerbæk M , et al. Circulating tumour DNA and circulating tumour cells in bladder cancer—From discovery to clinical implementation. Nat Rev Urol. 2025; 22( 9): 590– 608. doi:10.1038/s41585-025-01023-9. 40234713

[11] Herranz R , Oto J , Plana E , Pérez-Ardavín J , Verger P , Martínez-Sarmiento M , et al. Analysis of the fragmentation and integrity of urine cell-free DNA as a diagnostic and staging biomarker for bladder cancer. J Mol Diagn. 2025; 27( 12): 1189– 201. doi:10.1016/j.jmoldx.2025.08.010. 41016629

[12] Lu YT , Plets M , Morrison G , Cunha AT , Cen SY , Rhie SK , et al. Cell-free DNA methylation as a predictive biomarker of response to neoadjuvant chemotherapy for patients with muscle-invasive bladder cancer in SWOG S1314. Eur Urol Oncol. 2023; 6( 5): 516– 24. doi:10.1016/j.euo.2023.03.008. PMC10587361 37087309

[13] Wang B , Davis LE , Weight CJ , Abouassaly R , Bukavina L . Real-world experience with a commercial circulating tumor DNA assay in non–muscle-invasive bladder cancer. Eur Urol Oncol. 2025; 8( 4): 883– 7. doi:10.1016/j.euo.2025.05.019. 40517059

[14] Carrasco R , Ingelmo-Torres M , Trullas R , Roldán FL , Rodríguez-Carunchio L , Juez L , et al. Tumor-agnostic circulating tumor DNA testing for monitoring muscle-invasive bladder cancer. Int J Mol Sci. 2023; 24( 23): 16578. doi:10.3390/ijms242316578. PMC10706140 38068899

[15] Eraky A , Ben-David R , Hug B , Kolanukuduru KP , Almoflihi M , Waingankar N , et al. Bladder cancer with undetectable circulating tumor DNA after radical cystectomy may be amenable to a less intense imaging surveillance protocol: A diagnostic accuracy study. Eur Urol Oncol. 2025; 8( 6): 1505– 12. doi:10.1016/j.euo.2025.04.027. 40393820

[16] Vedeld HM , Pharo H , Sørbø AK , Brandt-Winge S , Five MB , Jeanmougin M , et al. Distinct longitudinal patterns of urine tumor DNA in patients undergoing surveillance for bladder cancer. Mol Oncol. 2024; 18( 11): 2684– 95. doi:10.1002/1878-0261.13639. PMC11547231 38720532

[17] Xue Z , Qie Y , Shen C , Wu Z , Chen H , Lin Y , et al. Urinary tumor DNA to identify candidates for repeat transurethral resection in non-muscle-invasive bladder cancer. Cancer Sci. 2026; 117( 3): 787– 96. doi:10.1111/cas.70311. PMC12951098 41445362

[18] Galsky MD , Izadmehr S , Yu M , Curtis SD , Douville C , Popoli M , et al. Monitoring of plasma and urine tumor-derived DNA to inform bladder-sparing approaches for patients with muscle-invasive bladder cancer. Proc Natl Acad Sci U S A. 2026; 123( 8): e2533449123. doi:10.1073/pnas.2533449123. PMC12933130 41706890

[19] Shi WY , Liu KJ , Esfahani MS , Mach KE , Phillips NA , Almanza D , et al. Field-effect-informed urine liquid biopsy for bladder cancer. Cell. 2026; 189( 4): 1024– 38.e9. doi:10.1016/j.cell.2025.12.054. PMC12962748 41605210

[20] Satyal U , Valentine H , Liu D , Slifker M , Lallas CD , Trabulsi EJ , et al. Urine biopsy as dynamic biomarker to enhance clinical staging of bladder cancer in radical cystectomy candidates. JCO Precis Oncol. 2024; 8: e2300362. doi:10.1200/PO.23.00362. PMC11671773 38865671

[21] Jeong IG , Yun SC , Ha HK , Kang SG , Lee S , Park S , et al. Urinary DNA methylation test for bladder cancer diagnosis. JAMA Oncol. 2025; 11( 3): 293– 9. doi:10.1001/jamaoncol.2024.6160. PMC11783243 39883469

[22] Vermeulen R , Bodinier B , Dagnino S , Wada R , Wang X , Silverman D , et al. A prospective study of smoking-related white blood cell DNA methylation markers and risk of bladder cancer. Eur J Epidemiol. 2024; 39( 4): 393– 407. doi:10.1007/s10654-024-01110-y. PMC11101379 38554236

[23] Komura K , Hirosuna K , Tokushige S , Tsujino T , Nishimura K , Ishida M , et al. The impact of FGFR3 alterations on the tumor microenvironment and the efficacy of immune checkpoint inhibitors in bladder cancer. Mol Cancer. 2023; 22( 1): 185. doi:10.1186/s12943-023-01897-6. PMC10657138 37980528

[24] Wu J , Lin Y , Yang K , Liu X , Wang H , Yu T , et al. Clinical effectiveness of a multitarget urine DNA test for urothelial carcinoma detection: A double-blinded, multicenter, prospective trial. Mol Cancer. 2024; 23( 1): 57. doi:10.1186/s12943-024-01974-4. PMC10949661 38504268

[25] Huang H , Liu A , Liang Y , Xin Y , Liu J , Hao Y , et al. A urinary assay for mutation and methylation biomarkers in the diagnosis and recurrence prediction of non-muscle invasive bladder cancer patients. BMC Med. 2023; 21( 1): 357. doi:10.1186/s12916-023-03065-5. PMC10510256 37726806

[26] Ramos P , Brás JP , Dias C , Bessa-Gonçalves M , Botelho F , Silva J , et al. Uromonitor: Clinical validation and performance assessment of a urinary biomarker within the surveillance of patients with nonmuscle-invasive bladder cancer. J Urol. 2025; 213( 3): 304– 12. doi:10.1097/JU.0000000000004335. 39561374

[27] Wolff I , Kravchuk AP , Wirtz RM , Schlomm T , Rabien A , Rong D , et al. Real-world performance of Uromonitor^®^ in urothelial bladder cancer detection: A multicentric trial. BJU Int. 2024; 134( 6): 992– 1000. doi:10.1111/bju.16450. 38923777

[28] Rubio-Briones J , Guerrero Ramos F , Mercadé Sánchez A , Bezana Abadía I , Rodríguez RM , Alcaraz A , et al. External validation of the Uromonitor^®^-version 2 urine test as a biomarker for optimisation of non-muscle-invasive bladder cancer management. BJU Int. 2026; 137( 1): 216– 24. doi:10.1111/bju.70010. 41031577

[29] Rabien A , Rong D , Rabenhorst S , Schlomm T , Labonté F , Hofbauer S , et al. Diagnostic performance of Uromonitor and TERTpm ddPCR urine tests for the non-invasive detection of bladder cancer. Sci Rep. 2024; 14( 1): 30617. doi:10.1038/s41598-024-83976-2. PMC11666540 39715826

[30] Dreyer T , Brandt S , Fabrin K , Azawi N , Vásquez JL , Ernst A , et al. Use of the xpert bladder cancer monitor urinary biomarker test for guiding cystoscopy in high-grade non–muscle-invasive bladder cancer: Results from the randomized controlled DaBlaCa-15 trial. Eur Urol. 2025; 88( 1): 23– 30. doi:10.1016/j.eururo.2025.03.018. 40280776

[31] Palermo M , D’elia C , Trenti E , Comploj E , Mian C , Schwienbacher C , et al. Prospective evaluation of the RT-PCR based urinary marker Bladder Epicheck^®^ as a diagnostic tool in upper urinary tract tumor. Minerva Urol Nephrol. 2024; 76( 2): 195– 202. doi:10.23736/s2724-6051.23.05488-5. 38498297

[32] Wang J , Guo F , Zhang J , Chao J . Potential-resolved electrochemiluminescence for simultaneous determination of multiplex bladder cancer markers. Chem Commun. 2024; 60( 34): 4609– 12. doi:10.1039/d4cc00996g. 38586987

[33] Arima J , Yoshino H , Fukumoto W , Kawahara I , Saito S , Li G , et al. LncRNA BCYRN1 as a potential therapeutic target and diagnostic marker in serum exosomes in bladder cancer. Int J Mol Sci. 2024; 25( 11): 5955. doi:10.3390/ijms25115955. PMC11172611 38892143

[34] Xu X , Chen J , Bai M , Liu T , Zhan S , Li J , et al. Plasma tsRNA signatures serve as a novel biomarker for bladder cancer. Cancer Sci. 2025; 116( 5): 1255– 67. doi:10.1111/cas.70003. PMC12044647 39948752

[35] Samara M , Vlachostergios PJ , Thodou E , Zachos I , Mitrakas L , Evmorfopoulos K , et al. Characterization of a miRNA signature with enhanced diagnostic and prognostic power for patients with bladder carcinoma. Int J Mol Sci. 2023; 24( 22): 16243. doi:10.3390/ijms242216243. PMC10671612 38003433

[36] Eckhart L , Rau S , Eckstein M , Stahl PR , Ayoubian H , Heinzelbecker J , et al. Machine learning accurately predicts muscle invasion of bladder cancer based on three miRNAs. J Cell Mol Med. 2025; 29( 3): e70361. doi:10.1111/jcmm.70361. PMC11810526 39929768

[37] Zhou H , Liu Q , Chen M , Xie Y , Xu W , Zhang X , et al. Urease-driven Janus nanomotors for dynamic enrichment and multiplexed detection of bladder cancer microRNAs in urine. ACS Sens. 2025; 10( 2): 1155– 65. doi:10.1021/acssensors.4c02996. 39907010

[38] Zhou Z , Zou L , Guan Y , Jiang L , Liu Y , Zhang X , et al. Survivin as a potential biomarker in the diagnosis of bladder cancer: A systematic review and meta-analysis. Urol Oncol. 2024; 42( 5): 133– 43. doi:10.1016/j.urolonc.2024.01.018. 38418270

[39] Pece A , Lovato G , Cela I , Mercatelli A , Ferro B , Nikkola J , et al. Glycosylated LGALS3BP is highly secreted by bladder cancer cells and represents a novel urinary disease biomarker. Mol Oncol. 2026; 20( 3): 823– 37. doi:10.1002/1878-0261.70140. PMC13042393 41046347

[40] Davalieva K , Kiprijanovska S , Ivanovski O , Trifunovski A , Saidi S , Dimovski A , et al. Proteomics profiling of bladder cancer tissues from early to advanced stages reveals NNMT and GALK1 as biomarkers for early detection and prognosis of BCa. Int J Mol Sci. 2023; 24( 19): 14938. doi:10.3390/ijms241914938. PMC10573217 37834386

[41] Kim M , Jung E , Song G , Joung J , Chung J , Seo H , et al. Diagnostic and prognostic potential of *SH3YL1* and *NOX4* in muscle-invasive bladder cancer. Int J Mol Sci. 2025; 26( 9): 3959. doi:10.3390/ijms26093959. PMC12071612 40362200

[42] Pagano I , Zhang Z , Luu M , Tikhonenkov S , Le Calvez-Kelm F , Goodison S , et al. Performance of the Oncuria-Detect bladder cancer test for evaluating patients presenting with haematuria: Results from a real-world clinical setting. J Transl Med. 2025; 23( 1): 680. doi:10.1186/s12967-025-06749-z. PMC12178018 40533776

[43] Keum C , Yeom H , Noh TI , Yi SY , Jin S , Kim C , et al. Diagnosis of early-stage bladder cancer via unprocessed urine samples at the point of care. Nat Biomed Eng. 2025; 9( 7): 1026– 38. doi:10.1038/s41551-024-01298-0. 39609560

[44] Maas M , Todenhöfer T , Black PC . Urine biomarkers in bladder cancer—Current status and future perspectives. Nat Rev Urol. 2023; 20( 10): 597– 614. doi:10.1038/s41585-023-00773-8. 37225864

[45] Teixeira-Marques A , Lourenço C , Oliveira MC , Henrique R , Jerónimo C . Extracellular vesicles as potential bladder cancer biomarkers: Take it or leave it? Int J Mol Sci. 2023; 24( 7): 6757. doi:10.3390/ijms24076757. PMC10094914 37047731

[46] Oeyen E , Hoekx L , De Wachter S , Baldewijns M , Ameye F , Mertens I . Bladder cancer diagnosis and follow-up: The current status and possible role of extracellular vesicles. Int J Mol Sci. 2019; 20( 4): 821. doi:10.3390/ijms20040821. PMC6412916 30769831

[47] Jordaens S , Oeyen E , Willems H , Ameye F , De Wachter S , Pauwels P , et al. Protein biomarker discovery studies on urinary sEV fractions separated with UF-SEC for the first diagnosis and detection of recurrence in bladder cancer patients. Biomolecules. 2023; 13( 6): 932. doi:10.3390/biom13060932. PMC10296596 37371512

[48] Steiner L , Eldh M , Offens A , Veerman RE , Johansson M , Hemdan T , et al. Protein profile in urinary extracellular vesicles is a marker of malignancy and correlates with muscle invasiveness in urinary bladder cancer. Cancer Lett. 2025; 609: 217352. doi:10.1016/j.canlet.2024.217352. 39586489

[49] Li Y , Fu B , Wang M , Chen W , Fan J , Li Y , et al. Urinary extracellular vesicle N-glycomics identifies diagnostic glycosignatures for bladder cancer. Nat Commun. 2025; 16( 1): 2292. doi:10.1038/s41467-025-57633-9. PMC11889218 40055327

[50] Sun N , Zhang Z , Yang X , Li J , Li Q , Kang J , et al. Unveiling urinary extracellular vesicle mRNA signature for early diagnosis and prognosis of bladder cancer. Theranostics. 2025; 15( 4): 1272– 84. doi:10.7150/thno.107213. PMC11729556 39816677

[51] Moreira IB , Rossdam C , Kaynert J , Beimdiek J , Vicente MM , Schmitz J , et al. Neolactotetraosylceramide enables urinary detection of bladder cancer. Cell Rep Med. 2025; 6( 8): 102246. doi:10.1016/j.xcrm.2025.102246. PMC12432384 40706589

[52] Wu N , Wong KY , Yu X , Zhao JW , Zhang XY , Wang JH , et al. Multispectral 3D DNA machine combined with multimodal machine learning for noninvasive precise diagnosis of bladder cancer. Anal Chem. 2024; 96( 24): 10046– 55. doi:10.1021/acs.analchem.4c01749. 38845359

[53] Chen H , Qi Y , Yang C , Tai Q , Zhang M , Shen XZ , et al. Heterogeneous MXene hybrid-oriented exosome isolation and metabolic profiling for early screening, subtyping and follow-up evaluation of bladder cancer. ACS Nano. 2023; 17( 23): 23924– 35. doi:10.1021/acsnano.3c08391. 38039354

[54] Cao Y , Feng J , Zhang Q , Deng C , Yang C , Li Y . Magnetic 3D macroporous MOF oriented urinary exosome metabolomics for early diagnosis of bladder cancer. J Nanobiotechnol. 2024; 22( 1): 671. doi:10.1186/s12951-024-02952-0. PMC11531116 39488699

[55] Li Q , Zhan S , Yang X , Zhang Z , Sun N , Wang X , et al. Choline phosphate-grafted nanozymes as universal extracellular vesicle probes for bladder cancer detection. ACS Nano. 2024; 18( 25): 16113– 25. doi:10.1021/acsnano.4c00280. 38857428

[56] Liu M , Jia G , Meng X , Rong Y , Xia Y , Hu Y , et al. Cyclic enrichment of urinary exosomes using a MOF-on-MOF-based asymmetric impinging streams chip for bladder cancer diagnosis and prognosis prediction. Adv Healthc Mater. 2025; 14( 14): 2500848. doi:10.1002/adhm.202500848. 40296360

[57] Wei X , Cai L , Li N , Fang Y , Wang J , Zhu Y . High-throughput screening of bladder cancer exosome biomarkers by barcodes integrated herringbone microfluidics. Biosens Bioelectron. 2026; 302: 118545. doi:10.1016/j.bios.2026.118545. 41740420

[58] Nizioł J , Ossoliński K , Płaza-Altamer A , Kołodziej A , Ossolińska A , Ossoliński T , et al. Untargeted urinary metabolomics for bladder cancer biomarker screening with ultrahigh-resolution mass spectrometry. Sci Rep. 2023; 13: 9802. doi:10.1038/s41598-023-36874-y. PMC10275937 37328580

[59] Liu Z , Teng C , Wan W , Wu F , Wu C , Ji W , et al. A panel of four plasma amino acids is a promising biomarker for newly diagnosed bladder cancer. Clin Nutr. 2024; 43( 7): 1599– 608. doi:10.1016/j.clnu.2024.05.003. 38776618

[60] Carapito Â , Fernandes Ferreira VS , Silva Ferreira AC , Teixeira-Marques A , Henrique R , Jerónimo C , et al. Non-invasive bladder cancer detection: Identification of a urinary volatile biomarker panel using GC-MS metabolomics and machine learning. Talanta. 2026; 297: 128749. doi:10.1016/j.talanta.2025.128749. 40907368

[61] Pal VK , Kannan K . Stability of volatile organic compound metabolites in urine at various storage temperatures and freeze-thaw cycles for 8 months. Environ Pollut. 2024; 345: 123493. doi:10.1016/j.envpol.2024.123493. PMC10939821 38316251

[62] Lu Y , Wang J , Bi X , Qian H , Pan J , Ye J . Non-invasive and rapid diagnosis of low-grade bladder cancer via SERSomes of urine. Nanoscale. 2025; 17( 12): 7303– 12. doi:10.1039/d4nr05306k. 39988954

[63] Sternberg CN , Bellmunt J , Sonpavde G , Siefker-Radtke AO , Stadler WM , Bajorin DF , et al. ICUD-EAU international consultation on bladder cancer 2012: Chemotherapy for urothelial carcinoma—Neoadjuvant and adjuvant settings. Eur Urol. 2013; 63( 1): 58– 66. doi:10.1016/j.eururo.2012.08.010. 22917984

[64] Bellmunt J , de Wit R , Vaughn DJ , Fradet Y , Lee JL , Fong L , et al. Pembrolizumab as second-line therapy for advanced urothelial carcinoma. N Engl J Med. 2017; 376( 11): 1015– 26. doi:10.1056/nejmoa1613683. PMC5635424 28212060

[65] Huang S , Huang Y , Li C , Liang Y , Huang M , Luo R , et al. Efficacy and safety of neoadjuvant PD-1 inhibitors or PD-L1 inhibitors for muscle invasive bladder cancer: A systematic review and meta-analysis. Front Immunol. 2024; 14: 1332213. doi:10.3389/fimmu.2023.1332213. PMC10803485 38264649

[66] Zuiverloon TCM , Nieuweboer AJM , Vékony H , Kirkels WJ , Bangma CH , Zwarthoff EC . Markers predicting response to bacillus calmette-guérin immunotherapy in high-risk bladder cancer patients: A systematic review. Eur Urol. 2012; 61( 1): 128– 45. doi:10.1016/j.eururo.2011.09.026. 22000498

[67] Funt SA , Rosenberg JE . Systemic, perioperative management of muscle-invasive bladder cancer and future horizons. Nat Rev Clin Oncol. 2017; 14( 4): 221– 34. doi:10.1038/nrclinonc.2016.188. PMC6054138 27874062

[68] Lindskrog SV , Birkenkamp-Demtröder K , Nordentoft I , Laliotis G , Lamy P , Christensen E , et al. Circulating tumor DNA analysis in advanced urothelial carcinoma: Insights from biological analysis and extended clinical follow-up. Clin Cancer Res. 2023; 29( 23): 4797– 807. doi:10.1158/1078-0432.CCR-23-1860. PMC10690087 37782315

[69] Nordentoft I , Lindskrog SV , Birkenkamp-Demtröder K , Gonzalez S , Kuzman M , Levatic J , et al. Whole-genome mutational analysis for tumor-informed detection of circulating tumor DNA in patients with urothelial carcinoma. Eur Urol. 2024; 86( 4): 301– 11. doi:10.1016/j.eururo.2024.05.014. 38811314

[70] Plimack ER , Tangen C , Plets M , Kokate R , Xiu J , Nabhan C , et al. Correlative analysis of ATM, RB1, ERCC2, and FANCC mutations and pathologic complete response after neoadjuvant chemotherapy in patients with muscle-invasive bladder cancer: Results from the SWOG S1314 trial. Eur Urol. 2024; 86( 4): 297– 300. doi:10.1016/j.eururo.2024.06.018. PMC11416320 39003201

[71] Gil-Jimenez A , van Dorp J , Contreras-Sanz A , van der Vos K , Vis DJ , Braaf L , et al. Assessment of predictive genomic biomarkers for response to cisplatin-based neoadjuvant chemotherapy in bladder cancer. Eur Urol. 2023; 83( 4): 313– 7. doi:10.1016/j.eururo.2022.07.023. 35965206

[72] Holmsten K , De Laere B , Sjödahl G , Lindberg J , Costa Svedman F , Östling P , et al. Exploring novel genomic biomarkers for response and survival after neoadjuvant chemotherapy and radical cystectomy of muscle-invasive bladder cancer. ESMO Open. 2025; 10( 8): 105512. doi:10.1016/j.esmoop.2025.105512. PMC12281969 40664147

[73] Olkhov-Mitsel E , Oberc A , Craddock KJ , Sherman C , Slodkowska E , Downes MR . MTAP protein status is highly concordant with *CDKN2A* fluorescent *in situ* hybridization and allows stratification of the luminal subtype in muscle-invasive bladder cancer. Histopathology. 2025; 86( 3): 352– 64. doi:10.1111/his.15324. PMC11707494 39327852

[74] Yan H , Ji X , Li B . Advancing personalized, predictive, and preventive medicine in bladder cancer: A multi-omics and machine learning approach for novel prognostic modeling, immune profiling, and therapeutic target discovery. Front Immunol. 2025; 16: 1572034. doi:10.3389/fimmu.2025.1572034. PMC12053186 40330458

[75] Pereira IOA , da Silva GN , Almeida TC , Lima APB , Sávio ALV , Leite KRM , et al. LncRNA *JHDM1D-AS1* is a key biomarker for progression and modulation of gemcitabine sensitivity in bladder cancer cells. Molecules. 2023; 28( 5): 2412. doi:10.3390/molecules28052412. PMC10005151 36903656

[76] He W , Xie J , Wang Z , Wang M , Chen Q , Zhang C , et al. Neoadjuvant treatment patterns and biomarker selection in muscle-invasive bladder cancer. Discov Oncol. 2025; 16( 1): 1197. doi:10.1007/s12672-025-02796-6. PMC12214084 40591027

[77] Zhao X , Yu X , Li W , Chen Z , Niu T , Weng X , et al. CDK6 as a biomarker for immunotherapy, drug sensitivity, and prognosis in bladder cancer: Bioinformatics and immunohistochemical analysis. Int J Med Sci. 2024; 21( 12): 2414– 29. doi:10.7150/ijms.101043. PMC11413897 39310261

[78] Yu Q , Xu S , Weng S , Ye L , Zheng H , Li D . GREM1 may be a biological indicator and potential target of bladder cancer. Sci Rep. 2024; 14( 1): 23280. doi:10.1038/s41598-024-73655-7. PMC11458565 39375386

[79] Trilla-Fuertes L , Pedregosa-Barbas J , García-Fernández E , Zambrana F , Martínez-Salas I , Gajate P , et al. Identification of a muscle-invasive bladder carcinoma molecular subtype of poor responders to neoadjuvant chemotherapy and high expression of targetable biomarkers. Int J Mol Sci. 2026; 27( 1): 476. doi:10.3390/ijms27010476. PMC12787041 41516353

[80] Holt MV , Dou Y , Young MN , Saltzman AB , Anurag M , Lei JT , et al. Proteogenomic characterization unveils biomarkers associated with chemoresistance in muscle-invasive bladder cancer. Cell Rep Med. 2025; 6( 8): 102255. doi:10.1016/j.xcrm.2025.102255. PMC12432383 40749681

[81] Giorgione R , Grasso D , Gambale E , Scolari F , Rossi V , Di Maida F , et al. Serum biomarkers in bladder cancer: NMR metabolomics for identification and monitoring during platinum-based therapy. Oncol Res. 2026; 34( 4): 1. doi:10.32604/or.2026.068896. PMC13040325 41930155

[82] Powles T , Assaf ZJ , Degaonkar V , Grivas P , Hussain M , Oudard S , et al. Updated overall survival by circulating tumor DNA status from the phase 3 IMvigor010 trial: Adjuvant atezolizumab versus observation in muscle-invasive urothelial carcinoma. Eur Urol. 2024; 85( 2): 114– 22. doi:10.1016/j.eururo.2023.06.007. 37500339

[83] Powles T , Kann AG , Castellano D , Gross-Goupil M , Nishiyama H , Bracarda S , et al. ctDNA-guided adjuvant atezolizumab in muscle-invasive bladder cancer. N Engl J Med. 2025; 393( 24): 2395– 408. doi:10.1056/NEJMoa2511885. 41124204

[84] Galsky MD , Bajorin DF , Tomita Y , Ye D , Agerbaek M , Enting D , et al. Adjuvant nivolumab in muscle-invasive urothelial carcinoma: Exploratory biomarker analysis of the randomized phase 3 CheckMate 274 trial. Nat Med. 2025; 31( 9): 3062– 73. doi:10.1038/s41591-025-03802-8. 40775055

[85] Klümper N , Eckstein M . Biomarkers of response to anti-NECTIN4 antibody-drug conjugate enfortumab vedotin in urothelial cancer. Eur Urol Focus. 2024; 10( 2): 224– 6. doi:10.1016/j.euf.2024.04.001. 38631991

[86] Klümper N , Tran NK , Zschäbitz S , Hahn O , Büttner T , Roghmann F , et al. *NECTIN4* amplification is frequent in solid tumors and predicts enfortumab vedotin response in metastatic urothelial cancer. J Clin Oncol. 2024; 42( 20): 2446– 55. doi:10.1200/JCO.23.01983. PMC11227306 38657187

[87] Nagata Y , Minato A , Aono H , Kimuro R , Higashijima K , Tomisaki I , et al. Immunohistochemical expression of p53 and FGFR3 predicts response to enfortumab vedotin in metastatic urothelial carcinoma. Int J Mol Sci. 2024; 25( 19): 10348. doi:10.3390/ijms251910348. PMC11477066 39408678

[88] Chiarugi P , Giannoni E . Anoikis: A necessary death program for anchorage-dependent cells. Biochem Pharmacol. 2008; 76( 11): 1352– 64. doi:10.1016/j.bcp.2008.07.023. 18708031

[89] Xie T , Peng S , Liu S , Zheng M , Diao W , Ding M , et al. Multi-cohort validation of Ascore: An anoikis-based prognostic signature for predicting disease progression and immunotherapy response in bladder cancer. Mol Cancer. 2024; 23( 1): 30. doi:10.1186/s12943-024-01945-9. PMC10858533 38341586

[90] Zhu L , Xiao F , Hou Y , Huang S , Xu Y , Guo X , et al. Identification of anoikis-related molecular patterns and the novel risk model to predict prognosis, tumor microenvironment infiltration and immunotherapy response in bladder cancer. Front Immunol. 2024; 15: 1491808. doi:10.3389/fimmu.2024.1491808. PMC11631915 39664392

[91] Zhang J , Wang Y , Yan Q , Wang H , Ran Q , Zhu H , et al. SWI/SNF complex alterations predict immunotherapy response in bladder cancer. Front Immunol. 2025; 16: 1708324. doi:10.3389/fimmu.2025.1708324. PMC12719508 41438758

[92] Nagumo Y , Hattori K , Kimura T , Sekino Y , Naiki T , Kobayashi Y , et al. Combined molecular subclass and immune phenotype correlate to atezolizumab plus radiation therapy response in invasive bladder cancer: BPT-ART phase 2 study. Int J Radiat Oncol. 2025; 122( 1): 168– 80. doi:10.1016/j.ijrobp.2024.12.019. 39755215

[93] Qi T , Yang W , Liu R , Deng D , Liu X . SWI/SNF-associated molecular subtypes reshape tumor microenvironmental features and inform precision therapeutic strategies in bladder cancer. Front Cell Infect Microbiol. 2026; 16: 1774929. doi:10.3389/fcimb.2026.1774929. PMC12957257 41789425

[94] Xu Z , Yang J , Ma Y , Tao B , He Y , Wu J , et al. Exploring of bladder cancer immune-related genes and potential therapeutic targets based on transcriptomic data and Mendelian randomization analysis. Front Immunol. 2025; 16: 1607098. doi:10.3389/fimmu.2025.1607098. PMC12313568 40755754

[95] Langfelder P , Lin ET , Tsai YT , Cha TL , Shieh GS . Gene signature for response prediction to immunotherapy and prognostic markers in metastatic urothelial carcinoma. Front Immunol. 2025; 16: 1607222. doi:10.3389/fimmu.2025.1607222. PMC12675356 41357226

[96] Yu W , Yao D , Ma X , Hou J , Tian J . A novel efferocytosis-related gene signature for predicting prognosis and therapeutic response in bladder cancer. Sci Rep. 2025; 15( 1): 19912. doi:10.1038/s41598-025-04037-w. PMC12144162 40481075

[97] Cao J , Zheng W . Angiogenesis-related gene NID2 profiling and immune infiltration in bladder cancer: Prognostic implications and immunotherapy response. Front Immunol. 2025; 16: 1615173. doi:10.3389/fimmu.2025.1615173. PMC12426854 40948763

[98] Peng Y , Song Y , Qin C , Ding M , Huang Z , Wang F , et al. Genomic subtypes of non-muscle-invasive bladder cancer: Guiding immunotherapy decision-making for patients exposed to aristolochic acid. Mol Med. 2025; 31( 1): 140. doi:10.1186/s10020-025-01199-1. PMC12004710 40247187

[99] Villoldo GM , Pombo MT , Aris M , Chemi J , Mandó P , Nagaraju S , et al. A Th2-score in the tumor microenvironment as a predictive biomarker of response to Bacillus Calmette Guérin in patients with non-muscle invasive bladder carcinoma: A retrospective study. Oncol Res. 2023; 31( 2): 207– 20. doi:10.32604/or.2023.028163. PMC10208021 37304240

[100] Francesca B , Meo M , Giudice FD , Scornajenghi CM , Gazzaniga P , Berardinis E , et al. Exploring the utility of a NGS multigene panel to predict BCG response in patients with non-muscle invasive bladder cancer. Oncol Res. 2025; 33( 3): 723– 31. doi:10.32604/or.2024.056282. PMC11915050 40109859

[101] Packiam VT , McElree IM , Ghodoussipour S , Nimgaonkar V , Krishna V , Kim JK , et al. Presence of an artificial intelligence-powered predictive biomarker is associated with a poor response to intravesical bacillus calmette-guerin but not to intravesical sequential gemcitabine/docetaxel in patients with high-grade non-muscle-invasive bladder cancer. Eur Urol Oncol. 2025; 8( 6): 1461– 5. doi:10.1016/j.euo.2025.04.006. PMC12907750 40287344

[102] Ben-David R , Tillu N , Cumarasamy S , Alerasool P , Rich JM , Kaufmann B , et al. Longitudinal tumor-informed circulating tumor DNA status predicts disease upstaging and poor prognosis for patients undergoing radical cystectomy. Eur Urol Oncol. 2024; 7( 5): 1105– 12. doi:10.1016/j.euo.2024.03.002. 38521660

[103] Sfakianos JP , Basu A , Laliotis G , Cumarasamy S , Rich JM , Kommalapati A , et al. Association of tumor-informed circulating tumor DNA detectability before and after radical cystectomy with disease-free survival in patients with bladder cancer. Eur Urol Oncol. 2025; 8( 2): 306– 14. doi:10.1016/j.euo.2024.07.001. 39013741

[104] Tasios A , Amstutz U , Seiler R , Fuhlbrück F , Oza N , Arnold N , et al. In patients with muscle-invasive bladder cancer undergoing radical cystectomy, dynamics of circulating tumor DNA following cystectomy: Association with patient outcomes. Eur Urol Focus. 2025; 11( 6): 959– 67. doi:10.1016/j.euf.2025.06.018. 40651904

[105] van Dorp J , Pipinikas C , Suelmann BBM , Mehra N , van Dijk N , Marsico G , et al. High- or low-dose preoperative ipilimumab plus nivolumab in stage III urothelial cancer: The phase 1B NABUCCO trial. Nat Med. 2023; 29( 3): 588– 92. doi:10.1038/s41591-022-02199-y. 36732628

[106] Li Y , Zhang L , Xu G , Xu G , Chen J , Zhao K , et al. Exploration and validation of a novel reactive oxygen species–related signature for predicting the prognosis and chemotherapy response of patients with bladder cancer. Front Immunol. 2024; 15: 1493528. doi:10.3389/fimmu.2024.1493528. PMC11693660 39749345

[107] Ma M , Wang S , Wang K , Jiang B , Li J , Hou S . CXCL12 links bladder cancer and diabetes as a potential biomarker. Sci Rep. 2025; 15: 19017. doi:10.1038/s41598-025-01357-9. PMC12125402 40447619

[108] Zhao Y , Xing Z , Zhao Y , Xu H , Liu R , Yang T , et al. Lactylation prognostic signature identifies DHCR7 as a modulator of chemoresistance and immunotherapy efficacy in bladder cancer. Front Immunol. 2025; 16: 1585727. doi:10.3389/fimmu.2025.1585727. PMC12303948 40735323

[109] Huang W , Xu Y , Liu J , Cheng T , Tang C . Identification and single-cell analysis of prognostic genes related to mitochondrial and neutrophil extracellular traps in bladder cancer. Sci Rep. 2025; 15( 1): 23982. doi:10.1038/s41598-025-10413-3. PMC12227698 40615671

[110] Du K , Kang N , Lin Y , Jia K , Shen C , Wu Z , et al. Senescence-associated signature based on immunotherapy response sequencing reveals PPIL3 as target for bladder cancer treatment and prognosis prediction. Front Immunol. 2025; 16: 1613056. doi:10.3389/fimmu.2025.1613056. PMC12240782 40642086

[111] Cai L , Zhang S , Zheng F , Ji F , Wang J , Shi L , et al. Identification of SCAMP2 as a regulator of NOTCH signaling in cisplatin resistance through a novel prognostic model for bladder cancer. Front Immunol. 2025; 16: 1573412. doi:10.3389/fimmu.2025.1573412. PMC12095277 40406117

[112] Zhang L , Gong Y , Chen J , Li M , Wang X , Wang W , et al. Prognostic significance of calcium signaling-related genes in bladder cancer and the role of ATP2B4 in regulating mitochondrial calcium ion levels via the VDAC1/MCU pathway. Front Immunol. 2026; 17: 1561666. doi:10.3389/fimmu.2026.1561666. PMC12920588 41727459

[113] Kim B , Jung M , Moon KC , Han D , Kim K , Kim H , et al. Quantitative proteomics identifies TUBB6 as a biomarker of muscle-invasion and poor prognosis in bladder cancer. Int J Cancer. 2023; 152( 2): 320– 30. doi:10.1002/ijc.34265. 36054443

[114] Jiang L , Sun G , Zou L , Guan Y , Hang Y , Liu Y , et al. Noncoding RNAs as a potential biomarker for the prognosis of bladder cancer: A systematic review and meta-analysis. Expert Rev Mol Diagn. 2023; 23( 4): 325– 34. doi:10.1080/14737159.2023.2195554. 36970945

[115] Wu Y , Liang J , Sun R , Liang Y , Li C , Zengin G , et al. Prognostic migrasome-associated long noncoding RNA model and tumor immune landscape in bladder cancer. J Cancer Metastasis Treat. 2026; 12: 8. doi:10.20517/2394-4722.2025.130.

[116] Han L , Yang H , Jiang X , Zhou Z , Ge C , Yu K , et al. Prognostic model based on disulfidptosis-related lncRNAs for predicting survival and therapeutic response in bladder cancer. Front Immunol. 2024; 15: 1512203. doi:10.3389/fimmu.2024.1512203. PMC11647029 39687628

[117] Cho M , Chang H , Kim JH . Integration of bulk and single-cell RNA-seq reveals prognostic gene signatures in patients with bladder cancer treated with immune checkpoint inhibitors. Cancer Immunol Immunother. 2024; 74( 1): 28. doi:10.1007/s00262-024-03839-7. PMC11663206 39708127

[118] Liu W , Wei C , He Q , Chen Z , Zhuang W , Guo Y , et al. Multiple omics integrative analysis identifies GARS1 as a novel prognostic and immunological biomarker: From pan-cancer to bladder cancer. Sci Rep. 2024; 14: 19025. doi:10.1038/s41598-024-70041-1. PMC11329754 39152248

[119] Yu QX , Wang JC , Liu JF , Ye LX , Guo YQ , Zheng HH . Adhesion-regulating molecule 1 (ADRM1) can be a potential biomarker and target for bladder cancer. Sci Rep. 2023; 13: 14803. doi:10.1038/s41598-023-41992-8. PMC10491834 37684377

[120] Li Y , Zhao B , Gao W , Wu Y , Tian T , Zhao S , et al. LDB2 is a novel diagnostic and prognostic biomarker and inhibits bladder cancer metastasis by activating p38 MAPK/ERK1/2/JNK signaling pathway. Clin Exp Med. 2025; 26( 1): 9. doi:10.1007/s10238-025-01854-1. PMC12628452 41249576

[121] Smolka C , Eckstein M , Jung R , Lieb V , Sikic D , Stöhr R , et al. Prognostic and predictive potential of CCL5 expression in muscle-invasive bladder cancer patients. Int J Mol Sci. 2024; 25( 12): 6325. doi:10.3390/ijms25126325. PMC11204343 38928033

[122] Vikerfors A , Davidsson S , Carlsson J , Jerlström T . Plasma levels of pentraxin 3: A potential prognostic biomarker in urinary bladder cancer patients. Int J Mol Sci. 2024; 25( 6): 3473. doi:10.3390/ijms25063473. PMC10970929 38542446

[123] Feng X , Zhang Z , Mao S . Prognostic and clinicopathological value of C-reactive protein in patients with bladder cancer: A meta-analysis. Ann Med. 2025; 57( 1): 2445781. doi:10.1080/07853890.2024.2445781. PMC11703067 39746669

[124] Hao Y , He W , Wang H , Rui W , Sun F , Zhu Y , et al. Forkhead box F1 functions as a novel prognostic biomarker and induces caspase-dependent apoptosis in bladder cancer. Oncol Rep. 2023; 50( 3): 173. doi:10.3892/or.2023.8610. PMC10433446 37539708

[125] Montironi R , Cimadamore A . Tumors of the urinary system and male genital organs: 2022 World Health Organization classification and multidisciplinarity. Eur Urol. 2022; 82( 5): 483– 6. doi:10.1016/j.eururo.2022.07.032. 35963651

[126] Lokeshwar VB , Habuchi T , Grossman HB , Murphy WM , Hautmann SH , Hemstreet GP , et al. Bladder tumor markers beyond cytology: International Consensus Panel on bladder tumor markers. Urology. 2005; 66( 6): 35– 63. doi:10.1016/j.urology.2005.08.064. 16399415

[127] Chauhan PS , Chen K , Babbra RK , Feng W , Pejovic N , Nallicheri A , et al. Urine tumor DNA detection of minimal residual disease in muscle-invasive bladder cancer treated with curative-intent radical cystectomy: A cohort study. PLoS Med. 2021; 18( 8): e1003732. doi:10.1371/journal.pmed.1003732. PMC8407541 34464379

